# Anthropometric Characteristics, Age, Sex, Drop Height, and Visual Feedback as Predictors of Dynamic Knee Valgus During Single-Leg Drop Landing

**DOI:** 10.3390/sports13050151

**Published:** 2025-05-19

**Authors:** Nuno Casanova, David Correia, Priscila Marconcin, Fábio Flôres, Denise Soares, Rodrigo Ruivo

**Affiliations:** 1 Insight: Piaget Research Center for Ecological Human Development, Instituto Piaget, 2805-059 Almada, Portugal; nuno.martins@ipiaget.pt (N.C.); priscila.marconcin@ipiaget.pt (P.M.); 2 Instituto Superior de Estudos Interculturais e Transdisciplinares de Almada, Instituto Piaget, 2805-059 Almada, Portugal; davidcorreia5@hotmail.com; 3 Faculty of Health Sciences, Universidad Autónoma de Chile, Providencia 7500912, Chile; 4 Centro de Investigação em Educação e Psicologia (CIEP), Universidade de Évora, 7004-516 Évora, Portugal; fabio.flores@uevora.pt; 5 Comprehensive Health Research Centre (CHRC), Universidade de Évora, 7004-516 Évora, Portugal; 6 Liberal Arts Department, American University of the Middle East, Egaila 54200, Kuwait; 7 Sport Physical Activity and Health Research & Innovation Center (SPRINT), 2040-413 Rio Maior, Portugal; rodrigoruivo@clinicadasconchas.pt

**Keywords:** knee stability, sensorimotor feedback, anatomical characteristics, movement screening, neuromuscular control, joint kinematics

## Abstract

The knee is a complex joint essential for locomotion, providing stability that is crucial for avoiding biomechanical deviations such as dynamic knee valgus (DKV), a contributing injury risk factor. This study aimed to assess the influence of body mass index (BMI), age, sex, anthropometric variables, visual feedback, and drop height on the occurrence of DKV. Forty healthy adults aged between 18 and 45 years, with a BMI between 18.5–29.9 kg/m^2^ and no lower limb injuries, were evaluated. Participants underwent a standardized warm-up, anthropometric measurements, and a single-leg drop-landing test from 20 to 30 cm, with and without visual feedback. Women exhibited significantly higher DKV in nearly all conditions. Statistically significant differences were observed between legs when no feedback was provided. Visual feedback significantly reduced DKV in one condition (left limb at 30 cm). Significant weak negative correlations with DKV were found for age, BMI, thigh length, and leg length. These data suggest that women may have higher DKV, anatomical variables may be associated with DKV, and visual feedback may have the potential to attenuate its occurrence. These findings highlight the importance of targeted interventions to attenuate DKV and underscore the role of body awareness and feedback in improving knee alignment.

## 1. Introduction

The knee joint is an important component of human locomotion, providing the necessary stability and mobility for activities ranging from walking to athletic endeavors, facilitating movements such as flexion and extension in the sagittal plane, and limited rotation [1]. Its role in distributing weight-bearing forces during dynamic movements underscores its importance in maintaining musculoskeletal health and preventing injuries. However, the knee is susceptible to biomechanical variations such as the dynamic knee valgus (DKV), characterized by a combination of internal rotation and adduction of the femur, abduction of the knee, anterior tibial translation, external tibial rotation, and ankle eversion [2]. While the etiology of knee injuries is multifactorial [3,4], DKV is associated with a higher risk of injury [5,6,7,8], although findings are not always consistent [9]. This altered alignment can lead to increased stress on the knee joint, particularly the anterior cruciate ligament (ACL), and has been associated with a higher risk of non-contact ACL injuries [10]. Additionally, DKV is linked to other knee pathologies, such as patellofemoral pain syndrome and tibiofemoral osteoarthritis [11]. The mechanism involves excessive frontal plane motion during dynamic tasks, which may overload knee structures and impair neuromuscular control [12]. Therefore, despite the unclear association between DKV and knee injury occurrence, understanding the biomechanical characteristics and modifiable and non-modifiable risk factors associated with DKV is paramount [13]. This comprehension is important for identifying individuals predisposed to knee injuries, enabling the implementation of targeted interventions aimed at reducing injury risk and enhancing movement quality during dynamic activities.

Understanding the multifactorial nature of DKV involves considering both modifiable and non-modifiable factors. Non-modifiable factors, such as age [10] and anatomical and anthropometrical variations [14], may influence the propensity for DKV. Moreover, anthropometric factors such as height and body mass index (BMI) are associated with DKV, with taller individuals [14] and those with higher BMI exhibiting greater DKV [15,16,17]. Additionally, sex differences have been observed in DKV prevalence, with females commonly presenting higher values compared to males [15,16,17]. These associations highlight the interplay between non-modifiable factors and DKV occurrence, underscoring the importance of considering individual characteristics in DKV risk assessment and intervention strategies. Conversely, modifiable factors, including muscle strength and activation, neuromuscular control, and ankle range of motion, have been previously linked to DKV [2]. For instance, a commonly proposed cause of DKV is weak hip abductors, extensors, and external rotators [18,19,20,21], although conflicting results have been found [22,23]. Furthermore, limited ankle dorsiflexion has also been shown to be associated with DKV [24]. However, the etiology of DKV is multifactorial, and other factors may also predispose an individual to this biomechanical variation. For instance, differences between dominant and non-dominant limbs have been observed [25], and it has also been shown that unilateral tasks may be accompanied by a higher degree of DKV in comparison to bilateral movements [26]. Furthermore, it could be hypothesized that higher drops, accompanied by higher landing forces, could be linked to higher degrees of DKV. Still, comparisons between drop heights have not been previously conducted.

While previous studies have predominantly focused on skeletal muscle function and anatomical characteristics, it could be postulated that DKV may also be influenced by exercise technique and body awareness. Feedback, particularly visual feedback, holds promise as a potentially valuable tool in addressing DKV by providing individuals with immediate information on their movement patterns, enabling individuals to observe and monitor their alignment and control in real-time, thereby offering opportunities for making adjustments to improve movement quality [27]. This real-time feedback mechanism can enhance kinesthetic awareness and proprioceptive control, allowing individuals to correct biomechanical deviations such as DKV actively. By facilitating a greater understanding of optimal movement patterns, visual feedback may aid in reducing the occurrence of DKV and promoting more efficient movement mechanics. However, although studies on the usage of visual feedback in correcting DKV are promising [28,29], they are currently limited.

Despite the recognized potential implications of DKV in predisposing individuals to knee injuries, the specific predictors contributing to its occurrence have yet to be fully understood. Moreover, it remains uncertain whether drop height affects DKV, and whether body awareness, mainly when guided by visual feedback, can effectively attenuate the incidence of DKV. The primary aim of this study was to investigate the influence of gender, anthropometric measurements, age, drop height, and visual feedback on the occurrence of dynamic knee valgus (DKV) during the single-leg drop-landing task, with a particular focus on comparing differences between males and females and evaluating the effectiveness of visual feedback in reducing DKV. It was hypothesized that anthropometric characteristics, age, sex, drop height, and visual feedback would influence the occurrence of dynamic knee valgus during single-leg drop-landing tasks, with higher drop heights leading to a greater occurrence of DKV, females presenting a greater DKV, and visual feedback expected to attenuate its occurrence.

## 2. Materials and Methods

### 2.1. Subjects

To determine the sample size, G*Power (v 3.1.9.7) [30] was used, with a significance level set at α = 0.05 and a desired power of 0.80. Based on an effect size of 0.64 reported in a prior study regarding the differences in DKV between dominant and non-dominant limbs [31], a sample size of 22 participants was calculated to conduct comparisons using paired *t*-tests. However, to accommodate additional comparisons between sexes, forty healthy adults aged 28.5 ± 7.6 years (19 males and 21 females) with a BMI of 23.5 ± 2.4 kg/m^2^ were recruited from an exercise clinic to participate in the present study. Inclusion criteria required individuals aged between 18 and 45 years, with a BMI ranging from 18.5 kg/m^2^ to 29.9 kg/m^2^, to be free of lower limb injuries at the time of assessment or within six months before the evaluation protocol application.

### 2.2. Procedures and Instruments

Eligible participants were identified through a questionnaire, and those who met the inclusion criteria received a thorough explanation of the research objectives and provided informed consent. Subsequently, a standardized warm-up was conducted to prepare participants for the testing protocol. This warm-up consisted of two sets of the following exercises: lunges (5 repetitions per leg), chair sit-to-stand (10 repetitions), and unilateral chair sit-to-stand (5 repetitions per leg, descending unilaterally and ascending bilaterally). These exercises were chosen to adequately warm up and prepare the participants’ lower bodies for the unilateral drop task.

Following the warm-up, anthropometric data (height, body mass, BMI, and segment lengths) were collected, and adhesive markers were applied for the DKV assessment. Participants then underwent an evaluation of DKV using the single-leg drop-landing task, during which they performed nine jumps on each leg. This included one familiarization repetition from a 20 cm height, two repetitions from a 20 cm height without feedback, two repetitions from a 30 cm height without feedback, and two repetitions from both 20 cm and 30 cm heights with visual feedback (observation of movement via a mirror). The mirror was placed approximately 2 m in front of the participants, allowing them to observe their movements in real-time during the task. The order of each condition (right or left leg and box height) was randomized. However, feedback conditions were performed after non-feedback trials. The drop heights of 20 cm and 30 cm were selected to induce controlled landing mechanics while minimizing injury risk, ensuring that the test was manageable for a non-athletic population and did not impose excessive biomechanical stress. This investigation was conducted following the principles outlined in the Declaration of Helsinki [31] and received approval from the University Ethics Committee (Reference: P02-S09-27042022).

### 2.3. Anthropometric Measurements

Body mass and height were measured using a SECA™ 761 mechanical professional scale measuring 303 × 118 × 470 mm (Bacelar & Irmão Lda, Porto, Portugal) and a SECA stadiometer measuring 337 × 2165 × 590 mm (GmBH & Co., Hamburg, Germany), respectively. Body mass index was calculated using the following formula: weight (kg)/height (m^2^). Segment lengths of the lower limbs were measured using a metric tape (with measurements in cm), where the distance between the anterior superior iliac spine and the medial malleolus was measured. This method is the clinical reference for measuring leg length discrepancy. A measurement was taken from the anterior superior iliac spine to the midpoint between the medial and lateral femoral condyles to measure thigh length [30]. In contrast, lower-leg length was measured from the midpoint between the medial and lateral femoral condyles to the medial malleolus.

### 2.4. Single-Leg Drop Landing—Dynamic Knee Valgus Assessment

Three adhesive markers were positioned for DKV assessment: one at the midpoint between the medial and lateral malleoli, another at the midpoint between the medial and lateral femoral condyles, and a third at the anterior-superior point between the knee joint and the anterior superior iliac spine.

The evaluation of DKV was conducted using the single-leg drop-landing test. The test involved unilateral landing from heights of 20 and 30 cm, with arms placed on the waist to eliminate arm swing during landing. During the single-leg drop-landing task, participants were instructed to keep the non-landing limb slightly flexed at the hip and knee to avoid contact with the ground and maintain balance throughout the landing phase. Visual inspection of the jumps was performed, and any discrepancies in the technique, the trial was considered invalid, and the subject was asked to repeat.

**Figure 1:**
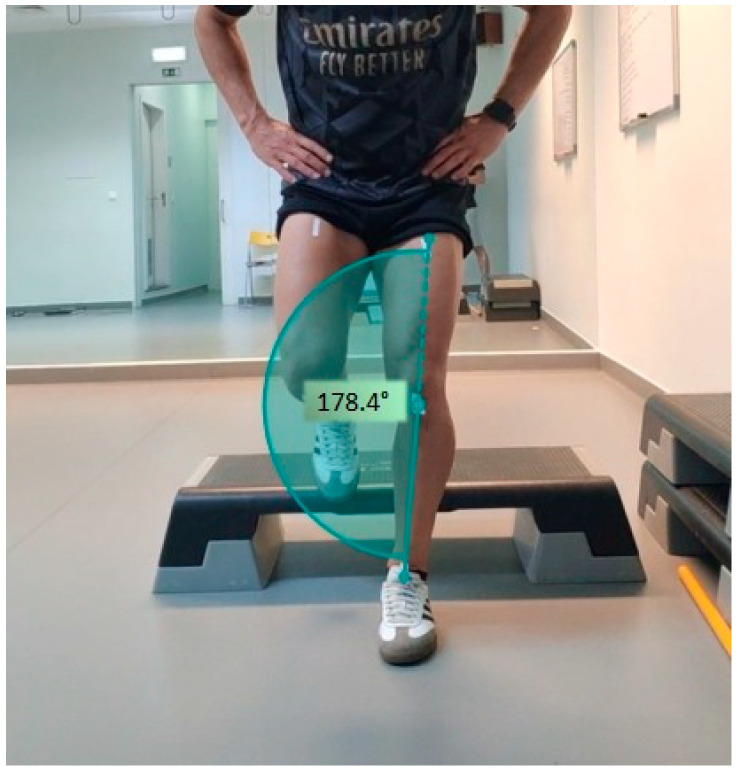
Angle measurement for DKV analysis.

Recordings were captured using a camera (Xiaomi, Beijing, China) stabilized on a tripod positioned 2 m away from the knee level, with a sample frequency of 120 Hz. The captured images and the calibration process were performed using the bio-photogrammetry software Kinovea (Kinovea.org) (Version 0.7.10). In the present investigation, the angle assessed is the projection angle in the frontal plane, determined by the straight line between the marked anatomical points, in which a knee angle exceeding 180 degrees indicates the presence of knee valgus (Figure 1) [14]. The angle was measured at the lowest point of the landing phase, where knee flexion was greatest, as this is the moment when DKV is most pronounced.

### 2.5. Statistical Analysis

The statistical analysis of the obtained data was conducted using SPSS software version 28 (IBM Corp., Armonk, New York, NY, USA). The Shapiro–Wilk and Levene’s tests were employed to examine the normal distribution and confirm the homogeneity of variances in the variables assessed in this study. Independent samples *t*-tests were used to compare the degree of DKV between males and females. Additionally, paired samples *t*-tests were utilized to compare jump heights, differences between the right and left limbs, and differences between the presence or absence of visual feedback. Pearson’s correlation test was used to analyze potential associations between anthropometric variables, age, and DKV. Correlation coefficients < 0.30 were considered weak, those between 0.30 and 0.70 were considered moderate, and coefficients > 0.70 were considered strong [32]. The data obtained from the single-leg drop-landing test represent the average of two jumps, with no statistically significant differences found between the two attempts (*p* > 0.05 for all comparisons). Data are presented as mean ± standard deviation, and statistical significance was set at *p* < 0.05.

## 3. Results

The descriptive characteristics for the entire sample, divided between men and women, can be observed in Table 1. As can be observed, there are statistically significant differences between men and women in all variables where men exhibited higher values, except for age and BMI.

Differences between Sexes

In Table 2, it is possible to observe the variables related to the single-leg drop-landing test to assess the presence of DKV for the entire sample and the differences between men and women. As observed from the table, statistically significant differences were found in all variables except for the variables ’Right—20 cm with Feedback’ and ’Left—30 cm with Feedback’. In the remaining variables and conditions, women presented higher DKV values than men.

Differences between the left and right side

In Table 3, it is possible to observe the variables related to the single-leg drop-landing test to assess the presence of DKV for the left and right limbs. As observed from the table data, statistically significant differences were found in two variables except for the ‘Right—20 cm with Feedback’ and ‘Left—30 cm with Feedback’. In the first two cases, higher DKV values were observed in the left limb compared to the right limb.

Differences between jump heights

Table 4 shows the variables related to the single-leg drop-landing test to assess the presence of DKV when performing the test at different heights (20 and 30 cm). As observed from the table data, no statistically significant differences were found between the two heights.

Differences between the presence or absence of visual feedback

In Table 5, it is possible to observe the variables related to the single-leg drop-landing test to assess the presence of DKV for the presence or absence of feedback (20 and 30 cm). As observed in the table, no statistically significant differences were found in the variables, except for the variable ’Left 30 cm’. In this case, visual feedback reduced the occurrence of DKV.

Correlations between anthropometric characteristics, age, and DKV

In Table 6, correlations between anthropometric characteristics and age with DKV can be observed. Several statistically significant correlations were observed regarding the right limb results. A moderate negative correlation was observed between age and DKV (in the 30 cm jump without feedback), where older participants exhibited lower DKV. In terms of BMI, a moderate negative correlation was found between BMI and DKV (in the 30 cm jump without feedback and in the 30 cm jump with feedback), with participants having higher BMI showing lower DKV. For Thigh Length, a negative correlation was observed between thigh length and DKV (in the 30 cm jump with feedback), where individuals with greater thigh length exhibited lower DKV. In the case of lower-limb length, a moderate negative correlation was observed between lower-limb length and DKV (in the 20 cm jump without feedback and in the 30 cm jump with feedback), where individuals with greater lower-limb length presented lower DKV. The remaining correlations were not statistically significant. Regarding the left limb, statistically significant correlations were observed between some variables. In the case of age, a moderate negative correlation was observed between age and DKV (in the 20 cm jump without feedback and in the 30 cm jump without feedback), where older participants exhibited lower DKV. For BMI, a moderate negative correlation was found between BMI and DKV (in the 30 cm jump without feedback), with participants having higher BMI showing lower DKV. The remaining correlations were not statistically significant (all *p* > 0.05).

## 4. Discussion

The primary aim of this study was to assess whether factors such as anthropometric measurements, age, sex, drop height, and visual feedback are associated with the occurrence of DKV during the single-leg drop-landing task. The results revealed significant differences between men and women across various conditions of the single-leg drop-landing test, in which, on average, women exhibited higher DKV values compared to men. Furthermore, statistically significant differences were observed in some conditions between the left and right sides, with higher DKV values observed in the left limb compared to the right limb. However, no statistically significant differences were found in DKV occurrence between different jump heights. Regarding the presence or absence of visual feedback, while no significant differences were found in most conditions, the presence of feedback was accompanied by a lower DKV occurrence in the left limb during the 30 cm jump condition.

The findings of this study reveal significant sex differences in the occurrence of DKV during the single-leg drop-landing task, in which females exhibited higher DKV values compared to males, particularly in conditions without visual feedback. This aligns with previous research indicating that females are more prone to valgus knee alignment during dynamic tasks, potentially due to differences in neuromuscular control, hip musculature strength, and anatomical characteristics such as a wider pelvis [15,16,17]. The increased DKV observed in females could predispose them to a higher risk of knee injuries, emphasizing the need for sex-specific intervention strategies aimed at improving knee stability and reducing injury risk [33].

In addition to sex differences, this study highlighted differences between the left and right limbs. In most conditions, the left limb consistently exhibited higher DKV values than the right limb. This asymmetry may be attributed to limb dominance, where the non-dominant limb often demonstrates weaker neuromuscular control and stability [34]. Limb dominance has also been shown to be related to the occurrence of DKV [25,35]. The observed asymmetry underscores the importance of incorporating unilateral exercises in training and rehabilitation programs to address imbalances and enhance overall lower limb stability. However, it is important to note that limb dominance was not assessed. In contrast, limb dominance could be related to the differences observed in the current study, particularly as only approximately 10–20% of the population preferentially uses the left limb [36]. The present data do not allow us to confirm this relationship confidently.

The influence of drop height on DKV was examined, revealing no significant differences between the 20 cm and 30 cm drop heights. This suggests that the drop height, within the range tested, does not substantially impact the degree of DKV during single-leg landings. While it could be postulated that differences would emerge, as different heights may induce different levels of forces impacting anatomic structures [37], a 10 cm difference between drop heights may not have been enough to observe statistically significant differences.

Visual feedback emerged as a potential modulator of DKV, particularly for the left limb during the 30 cm drop. Visual feedback significantly reduced DKV, indicating that real-time visual cues can enhance an individual’s awareness of knee alignment and promote corrective adjustments during dynamic tasks. Notably, the most significant decreases in DKV were observed in participants who initially exhibited higher deviation levels. This suggests visual feedback may be particularly effective for individuals with more pronounced DKV. Conversely, the absence of statistically significant differences in other conditions may be attributed to the already low levels of DKV, indicating minimal deviation to correct. This aligns with previous studies demonstrating the effectiveness of visual feedback in improving movement patterns and reducing biomechanical deviations [28]. These findings suggest that incorporating visual feedback mechanisms, such as mirrors or video monitoring, in training regimens may enhance proprioception and neuromuscular control.

Correlations between anthropometric characteristics, age, and DKV were also examined. Age demonstrated a moderate negative correlation with DKV, suggesting that older participants exhibited lower DKV values. This may be due to older participants potentially having more experience developing neuromuscular control, leading to improved stability during dynamic movements. However, the age range of the participants was relatively small (18–45 years old), and whether the same relationship would be maintained throughout aging and into the elderly population remains to be further examined. Additionally, a higher BMI was associated with lower levels of DKV, contrary to previous findings [14,38,39,40]. This inverse relationship might be explained by the higher stability provided by greater skeletal muscle during landing tasks or the absence of individuals with obesity, in whom biomechanical deviations have been more significantly observed [41]. Interestingly, thigh and lower-limb lengths were also negatively correlated with DKV, indicating that individuals with longer limbs exhibited lower degrees of knee valgus. However, similar to BMI, these correlations were weak and inconsistent. This suggests that, while anatomical characteristics may be associated with DKV, this biomechanical deviation may occur due to several factors, such as neuromuscular control, muscle strength, and proprioception.

The present study has limitations that should be acknowledged. Firstly, the investigation was limited to only two jump heights, which may restrict the conclusions that can be drawn. Different heights with varying gaps between them could potentially lead to more significant differences between conditions. Secondly, the absence of measurements for participants’ ankle range of motion and muscle strength restricts the comprehensive understanding of the factors influencing DKV occurrence. Furthermore, this study did not account for participants’ limb dominance, precluding the confirmation of whether the higher occurrence of DKV on the left limb was attributable to a higher prevalence of right limb dominance. Moreover, the overall low occurrence of DKV observed in the study population may have limited the effectiveness of feedback interventions, despite some individuals with higher DKV levels showing improvement with visual feedback. Future studies should explore a broader range of jump heights, incorporate assessments of joint range of motion and muscle strength, and conduct additional tests to understand DKV occurrence better. Additionally, investigating different feedback modalities may offer insights into practical strategies for DKV correction during dynamic tasks. Finally, it may be of interest to pre-select individuals exhibiting a higher degree of DKV to investigate whether various types of feedback can effectively attenuate its occurrence.

Understanding the factors influencing DKV during dynamic tasks such as single-leg drop landing is important for practitioners attempting to reduce the incidence of injuries and optimize performance. The findings from the present study suggest that sex differences may exist in DKV occurrence, with females exhibiting higher DKV values compared to males, which goes in agreement with previous studies [15,16,17]. Practitioners should be aware of these sex differences when designing training programs or interventions aimed at mitigating DKV-related injury risks. In this case, particular attention should be given to exercises in which a higher degree of DKV may occur, such as higher-impact unilateral tasks. Moreover, our study highlights the potential effectiveness of feedback interventions, particularly visual feedback, in reducing DKV occurrence. Therefore, apart from attempting to correct specific weaknesses (e.g., muscle strength, neuromuscular control, and joint range of motion), practitioners may consider integrating visual feedback techniques into training protocols to address DKV patterns and enhance movement quality. Additionally, future research exploring the impact of different types of feedback and interventions tailored to individuals with higher DKV levels could provide valuable insights for practitioners seeking to optimize movement biomechanics and reduce injury risks in their athletes or clients.

## 5. Conclusions

In conclusion, this study provides valuable insights into the factors influencing DKV occurrence during the single-leg drop-landing task, highlighting significant sex differences, limb asymmetries, and the potential role of visual feedback in reducing DKV. The findings suggest that females are more prone to exhibiting higher DKV values, which may contribute to an increased risk of knee injuries, emphasizing the need for targeted intervention strategies. Additionally, the observed asymmetry between the left and right limbs underscores the importance of addressing neuromuscular imbalances through unilateral training. While drop height did not significantly influence DKV, visual feedback demonstrated potential in improving knee alignment, particularly for individuals with pronounced deviations. Furthermore, weak correlations between anthropometric characteristics and DKV suggest that multiple biomechanical and neuromuscular factors contribute to its occurrence. Despite some limitations, such as the limited range of jump heights and the absence of limb dominance assessments, these findings offer practical implications for injury prevention and movement optimization. These findings suggest that caution is warranted, particularly for females, as they may exhibit higher DKV, which could potentially increase their risk of knee injuries. Additionally, visual feedback appears to be a promising approach for reducing DKV, particularly in conditions in which pronounced deviations are observed. Future research should explore a wider range of conditions, assess additional biomechanical variables, and investigate the effectiveness of different feedback interventions to enhance knee stability and mitigate injury risks.

## Figures and Tables

**Table 1:** The sample’s descriptive characteristics and differences between males and females.

Variables	Total (n = 40)	Male (n = 19)	Female (n = 21)	*p*-Value
Age (years)	28.5 ± 7.6	30.7 ± 8.5	26.6 ± 6.3	0.100
Body mass (kg)	67.0 ± 10.0	74.3 ± 6.8	60.0 ± 7.6	<0.001 *
Height (m)	1.68 ± 0.08	1.76 ± 0.04	1.62 ± 0.05	<0.001 *
Body mass index (kg/m^2^)	23.5 ± 2.4	24.1 ± 2.1	22.9 ± 2.6	0.142
Left lower leg cm)	38.8 ± 2.2	40.3 ± 1.5	37.4 ± 1.9	<0.001 *
Left thigh (cm)	49.2 ± 2.9	50.9 ± 2.8	47.7 ± 2.1	<0.001 *
Left lower limb (cm)	87.7 ± 4.8	91.0 ± 3.6	84.7 ± 3.7	<0.001 *
Right lower leg (cm)	38.4 ± 2.5	40.2 ± 1.7	36.8 ± 1.8	<0.001 *
Right thigh (cm)	49.3 ± 3.0	51.2 ± 2.6	47.6 ± 2.2	<0.001 *
Right lower limb (cm)	87.5 ± 4.9	90.9 ± 3.5	84.4 ± 3.9	<0.001 *

*—Statistically significant differences between males and females (*p* < 0.05).

**Table 2:** Dynamic knee valgus results during the single-leg drop-landing test for the entire (total) sample and comparison between sexes (male and female).

Variables	Total (n = 40)	Male (n = 19)	Female (n = 21)	*p*-Value
Right—20 cm without F	180.6 ± 8.1	179.1 ± 6.2	184.6 ± 5.1	<0.001 *
Right—30 cm without F	180.4 ± 8.2	176.6 ± 7.0	183.8 ± 7.8	0.004 *
Left—20 cm without F	182.7 ± 6.1	179.5 ± 5.1	185.6 ± 5.6	0.001 *
Left—30 cm without F	184.0 ± 7.6	179.7 ± 7.3	188.0 ± 5.5	<0.001 *
Right—20 cm with F	180.2 ± 7.1	178.7 ± 7.3	181.4 ± 6.9	0.240
Right—30 cm with F	180.1 ± 7.0	176.6 ± 6.4	183.3 ± 6.1	0.002 *
Left—20 cm with F	181.7 ± 6.6	179.4 ± 5.5	183.7 ± 7.0	0.036 *
Left—30 cm with F	180.2 ± 6.1	178.5 ± 6.5	181.8 ± 5.4	0.084

*—Statistically significant differences between men and women (*p* < 0.05). F—feedback.

**Table 3:** Dynamic knee valgus results during the single-leg drop-landing test comparison between members (left and right legs).

Variables	Right Leg	Left Leg	*p*-Value
20 cm without feedback	180.6 ± 8.1	182.7 ± 6.1	0.014 *
30 cm without feedback	180.4 ± 8.2	184.0 ± 7.6	0.001 *
20 cm with feedback	180.2 ± 7.1	181.7 ± 6.6	0.154
30 cm with feedback	180.1 ± 7.0	180.2 ± 6.1	0.938

*—Statistically significant differences between sides (*p* < 0.05).

**Table 4:** Dynamic knee valgus results during the single-leg drop-landing test comparison between jump heights (20 cm and 30 cm).

Variables	20 cm	30 cm	*p*-Value
Right without feedback	180.6 ± 8.1	180.4 ± 8.2	0.858
Left without feedback	182.7 ± 6.1	184.0 ± 7.6	0.146
Right with feedback	180.2 ± 7.1	180.1 ± 7.0	0.993
Left with feedback	181.7 ± 6.6	180.2 ± 6.1	0.116

**Table 5:** Dynamic knee valgus results during the single-leg drop-landing test: a comparison between conditions with and without feedback.

	With Feedback	Without Feedback	*p*-Value
Right 20 cm	180.2 ± 7.1	180.6 ± 8.1	0.715
Right 30 cm	180.1 ± 7.0	180.4 ± 8.2	0.821
Leg 20 cm	181.7 ± 6.6	182.7 ± 6.1	0.221
Left 30 cm	180.2 ± 6.1	184.0 ± 7.6	0.001 *

*—Statistically significant differences between the presence or absence of visual feedback (*p* < 0.05).

**Table 6:** Correlations between anthropometric characteristics, age, and DKV.

Variables			20 cm Without F	20 cm with F	30 cm Without F	30 cm with F
Right Limb	Age	R	−0.223	0.094	−0.376 *	−0.100
		*p*-value	0.166	0.563	0.017	0.540
	BMI	R	−0.276	−0.082	−0.446 *	−0.357 *
		*p*-value	0.085	0.613	0.004	0.024
	Lower-Leg Length	R	−0.312	−0.239	−0.248	−0.432
		*p*-value	0.050	0.138	0.122	0.005
	Thigh Length	R	−0.316	−0.103	−0.231	−0.360 *
		*p*-value	0.049	0.527	0.151	−0.023
	Lower-Limb Length	R	−0.316 *	−0.142	−0.299	−0.376 *
		*p*-value	0.047	0.384	0.061	0.017
Left Limb	Age	R	−0.316 *	−0.168	−0.353 *	−0.224
		*p*-value	0.047	0.299	0.025	0.165
	BMI	R	−0.277	−0.229	−0.430 *	−0.218
		*p*-value	0.083	0.155	0.006	0.176
	Lower-Leg Length	R	−0.207	−0.274	−0.232	−0.183
		*p*-value	0.200	0.087	0.150	0.259
	Thigh Length	R	−0.027	−0.086	−0.069	0.142
		*p*-value	0.868	0.596	0.674	0.382
	Lower-Limb Length	R	−0.162	−0.223	−0.161	−0.063
		*p*-value	0.319	0.166	0.321	0.698

*—Statistically significant correlations (*p* < 0.05).

## Data Availability

The original data presented in this study are openly available on zenodo.org at DOI: 10.5281/zenodo.15260698.

## Abbreviations

The following abbreviations are used in this manuscript:

DKV	Dynamic knee valgus
F	Feedback
BMI	Body mass index

## References

[1] Flandry F., Hommel G. (2011). Normal anatomy and biomechanics of the knee. Sports Med. Arthrosc. Rev..

[2] Wilczyński B., Zorena K., Ślęzak D. (2020). Dynamic Knee Valgus in Single-Leg Movement Tasks. Potentially Modifiable Factors and Exercise Training Options. A Literature Review. Int. J. Environ. Res. Public Health.

[3] Yung K.K., Ardern C.L., Serpiello F.R., Robertson S. (2022). Characteristics of Complex Systems in Sports Injury Rehabilitation: Examples and Implications for Practice. Sports Med. Open.

[4] Schweizer N., Strutzenberger G., Franchi M.V., Farshad M., Scherr J., Spörri J. (2022). Screening Tests for Assessing Athletes at Risk of ACL Injury or Reinjury-A Scoping Review. Int. J. Environ. Res. Public Health.

[5] Grassi A., Smiley S.P., Roberti di Sarsina T., Signorelli C., Marcheggiani Muccioli G.M., Bondi A., Romagnoli M., Agostini A., Zaffagnini S. (2017). Mechanisms and situations of anterior cruciate ligament injuries in professional male soccer players: A YouTube-based video analysis. Eur. J. Orthop. Surg. Traumatol..

[6] Dinis R., Vaz J.R., Silva L., Marta S., Pezarat-Correia P. (2021). Electromyographic and kinematic analysis of females with excessive medial knee displacement in the overhead squat. J. Electromyogr. Kinesiol..

[7] Holden S., Boreham C., Delahunt E. (2016). Sex Differences in Landing Biomechanics and Postural Stability During Adolescence: A Systematic Review with Meta-Analyses. Sports Med..

[8] Waldén M., Krosshaug T., Bjørneboe J., Andersen T.E., Faul O., Hägglund M. (2015). Three distinct mechanisms predominate in non-contact anterior cruciate ligament injuries in male professional football players: A systematic video analysis of 39 cases. Br. J. Sports Med..

[9] Nilstad A., Petushek E., Mok K.M., Bahr R., Krosshaug T. (2023). Kiss goodbye to the ‘kissing knees’: No association between frontal plane inward knee motion and risk of future non-contact ACL injury in elite female athletes. Sports Biomech..

[10] Numata H., Nakase J., Kitaoka K., Shima Y., Oshima T., Takata Y., Shimozaki K., Tsuchiya H. (2018). Two-dimensional motion analysis of dynamic knee valgus identifies female high school athletes at risk of non-contact anterior cruciate ligament injury. Knee Surg. Sports Traumatol. Arthrosc..

[11] Norasteh A.A., Fadaei Dehcheshmeh M., Shamlou Kazemi A. (2023). The Role of Dynamic Knee Valgus in Occurrence of Knee Injuries: A Review Study. Sci. J. Rehabil. Med..

[12] Yalfani A., Ahmadi M., Asgarpoor A. (2024). The effect of kinetic factors of dynamic knee valgus on patellofemoral pain: A systematic review and meta-analysis. J. Bodyw. Mov. Ther..

[13] Boyer K.A., Andriacchi T.P. (2016). The Nature of Age-Related Differences in Knee Function during Walking: Implication for the Development of Knee Osteoarthritis. PLoS ONE.

[14] Asaeda M., Nakamae A., Hirata K., Kono Y., Uenishi H., Adachi N. (2020). Factors associated with dynamic knee valgus angle during single-leg forward landing in patients after anterior cruciate ligament reconstruction. Asia Pac. J. Sports Med. Arthrosc. Rehabil. Technol..

[15] Ford K.R., Myer G.D., Hewett T.E. (2003). Valgus knee motion during landing in high school female and male basketball players. Med. Sci. Sports Exerc..

[16] Russell K.A., Palmieri R.M., Zinder S.M., Ingersoll C.D. (2006). Sex differences in valgus knee angle during a single-leg drop jump. J. Athl. Train..

[17] Schmitz R.J., Shultz S.J., Nguyen A.D. (2009). Dynamic valgus alignment and functional strength in males and females during maturation. J. Athl. Train..

[18] Claiborne T.L., Armstrong C.W., Gandhi V., Pincivero D.M. (2006). Relationship between hip and knee strength and knee valgus during a single leg squat. J. Appl. Biomech..

[19] Hollman J.H., Ginos B.E., Kozuchowski J., Vaughn A.S., Krause D.A., Youdas J.W. (2009). Relationships between knee valgus, hip-muscle strength, and hip-muscle recruitment during a single-limb step-down. J. Sport Rehabil..

[20] Stickler L., Finley M., Gulgin H. (2015). Relationship between hip and core strength and frontal plane alignment during a single leg squat. Phys. Ther. Sport.

[21] Suzuki H., Omori G., Uematsu D., Nishino K., Endo N. (2015). The influence of hip strength on knee kinematics during a single-legged medial drop landing among competitive collegiate basketball players. Int. J. Sports Phys. Ther..

[22] Cashman G.E. (2012). The effect of weak hip abductors or external rotators on knee valgus kinematics in healthy subjects: A systematic review. J. Sport Rehabil..

[23] Dix J., Marsh S., Dingenen B., Malliaras P. (2019). The relationship between hip muscle strength and dynamic knee valgus in asymptomatic females: A systematic review. Phys. Ther. Sport.

[24] Lima Y.L., Ferreira V., de Paula Lima P.O., Bezerra M.A., de Oliveira R.R., Almeida G.P.L. (2018). The association of ankle dorsiflexion and dynamic knee valgus: A systematic review and meta-analysis. Phys. Ther. Sport.

[25] Ludwig O., Simon S., Piret J., Becker S., Marschall F. (2017). Differences in the Dominant and Non-Dominant Knee Valgus Angle in Junior Elite and Amateur Soccer Players after Unilateral Landing. Sports.

[26] Munro A., Herrington L., Comfort P. (2017). The Relationship Between 2-Dimensional Knee-Valgus Angles During Single-Leg Squat, Single-Leg-Land, and Drop-Jump Screening Tests. J. Sport Rehabil..

[27] Mödinger M., Woll A., Wagner I. (2022). Video-based visual feedback to enhance motor learning in physical education—A systematic review. Ger. J. Exerc. Sport Res..

[28] Marshall A.N., Hertel J., Hart J.M., Russell S., Saliba S.A. (2020). Visual Biofeedback and Changes in Lower Extremity Kinematics in Individuals with Medial Knee Displacement. J. Athl. Train..

[29] Shams F., Hadadnezhad M., Letafatkar A., Hogg J. (2022). Valgus Control Feedback and Taping Improves the Effects of Plyometric Exercises in Women with Dynamic Knee Valgus. Sports Health.

[30] Faul F., Erdfelder E., Lang A.-G., Buchner A. (2007). G* Power 3: A flexible statistical power analysis program for the social, behavioral, and biomedical sciences. Behav. Res. Methods.

[31] (2014). World Medical Association Declaration of Helsinki: Ethical principles for medical research involving human subjects. J. Am. Coll. Dent..

[32] Field A. (2013). Discovering Statistics Using IBM SPSS Statistics.

[33] Sahabuddin F.N.A., Jamaludin N.I., Amir N.H., Shaharudin S. (2021). The effects of hip- and ankle-focused exercise intervention on dynamic knee valgus: A systematic review. PeerJ.

[34] Virgile A., Bishop C. (2021). A Narrative Review of Limb Dominance: Task Specificity and the Importance of Fitness Testing. J. Strength Cond. Res..

[35] Alfuth M., Fichter P., Knicker A. (2021). Leg length discrepancy: A systematic review on the validity and reliability of clinical assessments and imaging diagnostics used in clinical practice. PLoS ONE.

[36] Hansford K.J., Baker D.H., McKenzie K.J., Preston C.E.J. (2024). Multisensory processing and proprioceptive plasticity during resizing illusions. Exp. Brain Res..

[37] Bi G., Hua L., Sun J., Xu Q., Li G. (2024). Impact of different landing heights on the contact force in the medial tibiofemoral compartment and the surrounding muscle force characteristics in drop jumps. PLoS ONE.

[38] Bout-Tabaku S., Shults J., Zemel B.S., Leonard M.B., Berkowitz R.I., Stettler N., Burnham J.M. (2015). Obesity is associated with greater valgus knee alignment in pubertal children, and higher body mass index is associated with greater variability in knee alignment in girls. J. Rheumatol..

[39] Soheilipour F., Pazouki A., Mazaherinezhad A., Yagoubzadeh K., Dadgostar H., Rouhani F. (2020). The Prevalence of Genu Varum and Genu Valgum in Overweight and Obese Patients: Assessing the Relationship between Body Mass Index and Knee Angular Deformities. Acta Biomed..

[40] Taylor E.D., Theim K.R., Mirch M.C., Ghorbani S., Tanofsky-Kraff M., Adler-Wailes D.C., Brady S., Reynolds J.C., Calis K.A., Yanovski J.A. (2006). Orthopedic complications of overweight in children and adolescents. Pediatrics.

[41] Capodaglio P., Gobbi M., Donno L., Fumagalli A., Buratto C., Galli M., Cimolin V. (2021). Effect of Obesity on Knee and Ankle Biomechanics during Walking. Sensors.

